# Innovations, Challenges and Future Directions of T7RNA Polymerase in Microbial Cell Factories

**DOI:** 10.1021/acssynbio.5c00139

**Published:** 2025-04-10

**Authors:** Sefli Sri Wahyu Effendi, I-Son Ng

**Affiliations:** Department of Chemical Engineering, National Cheng Kung University, Tainan 701, Taiwan

**Keywords:** T7RNAP, gene regulation, circuit design, genetic engineering, microbial cell factory

## Abstract

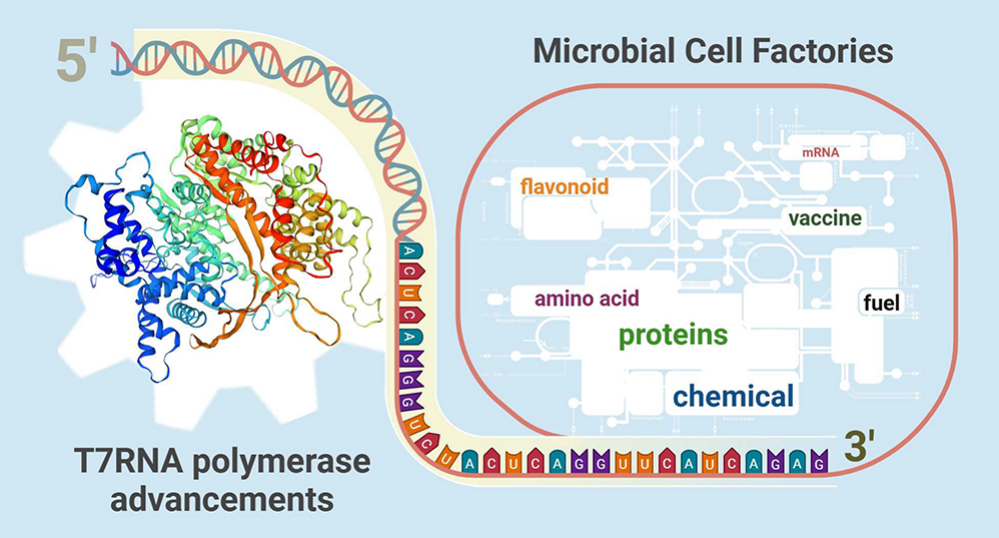

The study of “resource allocator” bacteriophage T7 RNA polymerase (T7RNAP) has garnered significant interest, particularly for optimizing transcriptional systems in microbial cell factories (MCFs). Most previous reviews have primarily focused on T7RNAP by dissecting specific aspects of its molecular structure and functional dynamics; this critical review seeks to broaden the scope. We emphasize a comprehensive guide in utilizing the versatile T7RNAP variants, covering both fundamental principles and fine-tuned circuit designs for synthetic biology applications. Recent advancements in engineered T7RNAP with enhanced specificity and controllability are also highlighted. Furthermore, we discuss the host compatibility considerations for implementing T7RNAP systems in sustainable bioproduction. Finally, key challenges of regulatory complexities and emerging opportunities for next-generation T7RNAP technology are discussed, reinforcing future directions for improving MCF performance.

## 1 Introduction

Microbial cell factories have been urged as a promising alternative to conventional chemical processes, meeting sustainable needs. However, in most cases, the wild-type hosts are still inadequate in overproducing desired metabolites due to poor enzyme activities, stress tolerance, and other intolerable properties. Over the decades, synthetic biology has emerged to genetically engineer the biological elements in replication, transcription, and translation of the host cell, thus allowing for optimum control of genetic circuits and cellular functions. RNA Polymerase (RNAP) is the main component of transcription machinery and serves as a “resource allocator” of metabolic fluxes.^1,2^ RNAP also participates in many biochemical events, especially cell growth and adaptation to an ever-changing cellular milieu.^1,3^ Of RNAP sources, bacteriophage T7RNAP is known to be one of the simplest polymerases in RNA synthesis because it consists of a single subunit without additional protein factors. The most distinctive feature of T7RNAP is its specificity for the T7 promoter, allowing precise control over the DNA sequences downstream of it.^4^ Compared to native RNAP in *Escherichia coli*, bacteriophage T7RNAP exhibits a 5-fold transcriptional rate.^5^ Moreover, the T7 promoter can produce long transcripts, which are beneficial for expressing polycistronic genes, especially in gene clusters of natural products.^6^

**Figure 1:**
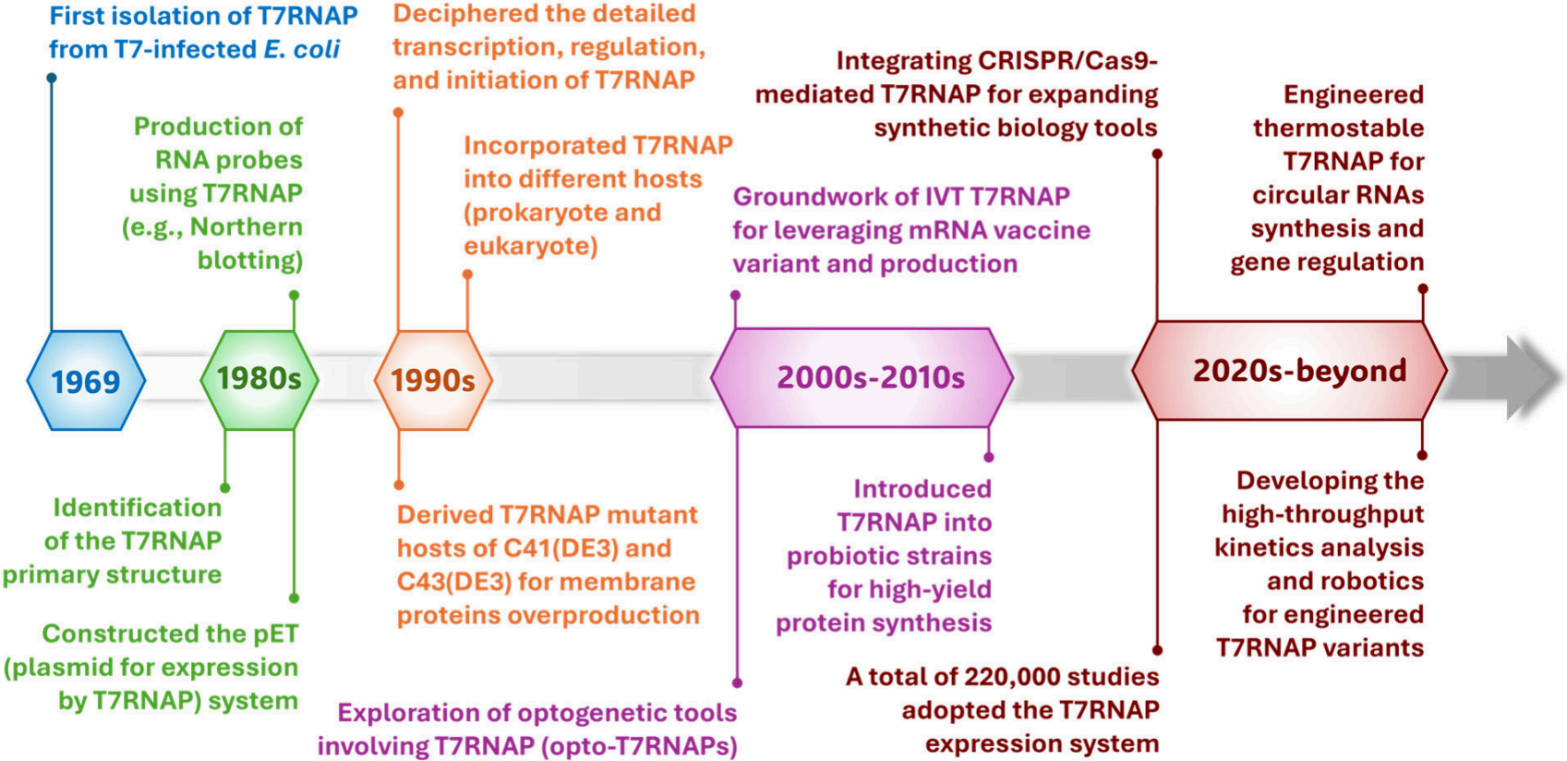
Key milestones of T7RNAP from first isolation to recent breakthroughs.

A series of significant milestones, from the discovery of the bacteriophage T7RNAP in 1969 to key breakthroughs in the 21st century, is presented in Figure 1. T7RNAP was first isolated from T7-infected *E. coli* cells in 1969.^5^ The primary structure of T7RNAP was identified using X-ray crystal analysis during the 1980s, thus successfully presenting the model of its initiation complex with N-terminal domain (NTD) and polymerase domain.^7,8^ NTD residues from 1 to 310 bp correspond to specific recognition and binding to the promoter. Meanwhile, the polymerase domain consists of three subdomains: thumb (aa ∼ 330–410), palm (aa 386–838), and fingers (aa 541–778). Each subdomain stands for stabilizing a clamp upon binding to DNA to prevent complex dissociation, catalyzing RNA synthesis, and assisting the correct position of ribonucleoside triphosphates (rNTPs) into RNA strands.^9,10^ T7RNAP complex undergoes a conformational change to ensure efficient and accurate DNA transcription into RNA initiation.^11−14^ As a result, a tight regulation control of T7RNAP was also exploited to develop a powerful pET system (plasmid for expression by T7RNAP), which became popular for producing high-yield recombinant proteins and expressing toxic proteins.^15^

Numerous impressive studies of T7RNAP over the past 20 years focused on exploration and utilization, including T7RNAP transcription-based development *in vivo* across a broad range of hosts and *in vitro* as a cell-free system.^9,10^ The applications have broadened into optogenetic tools for revolutionizing gene control under a multiwavelength system. Recently, *in vitro* transcription has been used to accelerate mRNA vaccine production and probiotic hosts for safer synthesis of desired proteins.^16−20^ As a cornerstone in synthetic biology, 220,000 works utilizing the T7RNAP expression system were recorded in the early 2020s.^21^ Among all, a vital exploration of T7RNAP was noted through its incorporation with the CRISPR system (i.e., Clustered Regularly Interspaced Short Palindromic Repeats) and Cas9 enzyme to enhance gene editing efficiency. This combination could ensure a sufficient supply of guide RNAs and direct the Cas9 to the correct genomic point, thus hindering the risk of off-target effects and escape rates from Cas9 cleavage.^22,23^ Moreover, T7RNAP has been successfully harnessed for *in vivo* directed evolution applications. Among the notable projects, the evolution of the T7 system could generate random mutagenesis by using T7RNAP fused with base deaminases, such as MutaT7 tools and T7-DIVA (T7-targeted dCas9-limited *in vivo* mutagenesis) platform in *E. coli*, also TRIDENT (TaRgeted *In vivo*Diversification ENabled by T7RNAP) in broad cells.^24−26^ Another advancement was made by engineering thermostable T7RNAP to withstand higher temperatures, streamlining with efficient cotranscriptional capping, double-stranded RNA (dsRNA), and circular RNA synthesis.^27,28^ Recently, high-throughput kinetic analysis of T7RNAP has been integrated with robotics, generating HiKER (High-throughput Kinetics using Capillary Electrophoresis and Robotics) to significantly enhance the efficiency and accuracy of enzyme kinetic studies, which could collect 1500 points in a single workday and hinder RNAP misincorporation under various conditions^29^ (Figure 1). Finally, the fine-tuned T7RNAP variants have improved transcription efficiency and reduced error rates in cell factories.

Previous reviews have covered T7RNAP in great detail;^9,10,30^ however, profound progress continues to be created in developing robust microbial cell factories (MCFs). This review highlights both foundational and advanced strategies for employing T7RNAP in MCFs, addressing key questions that will come in handy before dealing with the circuit design. The challenges and opportunities are critically discussed, offering guidance for the future development of efficient and versatile T7RNAP variants toward MCF applications.

## 2 When is T7RNAP Used *In Vivo* or *In Vitro*?

**Figure 2:**
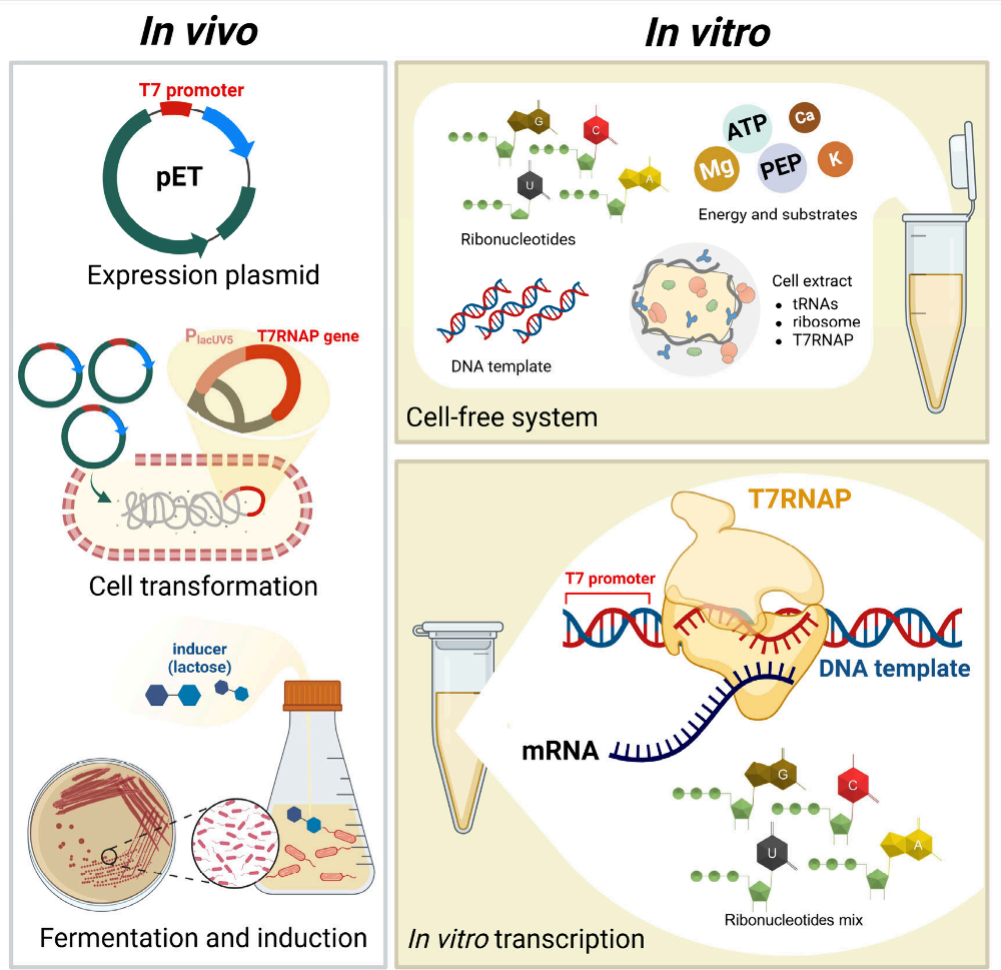
Illustration of T7RNAP usage *in vivo* for recombinant protein production and *in vitro* for chemical biosynthesis using the cell-free system and mRNA synthesis using the IVT tool.

T7RNAP and T7 orthogonality can be applied either *in vivo* or *in vitro* with different techniques (Figure 2). T7RNAP is commonly used in living bacterial hosts like *E. coli* to produce large amounts of recombinant proteins. The gene of interest (GOI) is cloned into a plasmid under the control of a T7 promoter, while T7RNAP is provided by involving either the chromosome-integrated T7RNAP or the plasmid-driven T7RNAP. To control the orthogonality level and GOI expression tightly, T7RNAP is mostly regulated by inducible promoters when enzymes or products are toxic to the host if expressed continuously.^10,31,32^*In vivo* works are mostly used to (i) enable dynamic control of gene expression that responds to specific metabolites based on cellular conditions. For instance, T7RNAP could function as a ligand-activated RNA polymerase in which gene expression was modulated by relevant concentrations of indoles;^33^ (ii) link with quorum sensing (QS) pathways for regulating gene expression in response to cell density. When the cell population reaches a threshold density, QS signals could trigger T7RNAP activity, initiating group-wide behaviors to synthesize desired enzymes or metabolites;^34,35^ or (iii) act as biosensor compartments. As an example, T7RNAP transcription incorporated with TbuT transcriptional regulator from *Ralstonia pickettii* enhanced biosensor sensitivity even at the low levels of isoprene.^36,37^

Beyond cells, T7RNAP is widely utilized for recombinant protein and RNA synthesis, known as the cell-free system (CFS) and *in vitro* transcription (IVT), respectively.^38,39^ Both applications are conducted by simply mixing the components and then incubating the reaction at the defined conditions. CFS uses cellular components of ribosomes, tRNAs, and enzymes extracted from cells to conduct biological reactions in a test tube,^40^ while IVT requires a purified DNA template, T7RNAP, nucleotides, suitable cofactors, and buffer conditions. The reaction mixture is subsequently incubated at an optimal temperature (usually at 37 °C).^39,41^ The DNA template is prepared using linearized plasmid or PCR product that contains antigen sequences, 5′- and 3′-UTRs, and a T7 promoter upstream of the 5′-UTR. T7RNAP recognizes the T7 promoter of the DNA template and initiates mRNA transcription.^39,42^

CFS is suggested as a powerful alternative to a classical *in vivo* system by neglecting the complexity of living cells for producing inclusion bodies, toxic proteins, protease-prone proteins, and isotope-labeled amino acid proteins.^43−45^ In the early CFS exploration, the T7 system could synthesize a high eGFP protein with a yield of 2.3 mg/mL.^46,47^ The powerful system of CFS also has achieved great success in synthesizing different types of macromolecules, such as immunoglobulins^48^ and membrane proteins,^49^ and even complexes, such as *E. coli* ribosome^50,51^ and RNA virus.^52^ To scale CFS for industrial manufacture is a challenge via an *in vivo* approach, but utilizing the T7 system for CFS still makes it a powerful tool for accelerating research progress and understanding complex biological processes. Furthermore, IVT using T7RNAP successfully created an important milestone by producing the COVID-19 mRNA vaccine, since T7RNAP could cotranscriptionally incorporate with the modified nucleotides (e.g., pseudouridine, as a substrate of COVID-19 vaccines) and turn into RNA, allowing for internal RNA modifications and reducing immunogenicity.^38,39^ Aside from its ability to incorporate noncanonical nucleotides, T7RNAP is capable of capping with various cofactors, such as NAD^+^, with up to 50% efficiency *in vitro*, NADH, FAD, and coenzyme A derivatives.^27,38^ IVT mRNA production has also been considered more efficient for synthesizing long transcripts (>kilobases in length) with high yield than using chemically synthesized RNA.^39,53,54^ In response to the rapid advancements in RNA vaccines and therapeutics, the importance of IVT using T7RNAP has also expanded and accounted for 34% of the overall raw material cost for mRNA manufacturing.^55^ However, during the IVT process, T7RNAP also produces immunostimulatory byproducts such as double-stranded RNA (dsRNA) that can affect mRNA purity and effectiveness. Hence, rational-engineered T7RNAP is necessary to simplify the downstream process with similar mRNA potency and lower immunostimulatory content.^56,57^ Taken together, the decision to use T7RNAP *in vivo* or *in vitro* depends on the specific goals that *in vivo* excels in dynamic gene expression and biosensing, while *in vitro* offers simplicity for efficient protein and RNA synthesis. Both approaches complement MCF applications.

## 3 How to Design the T7RNAP Orthogonality System?

**Figure 3:**
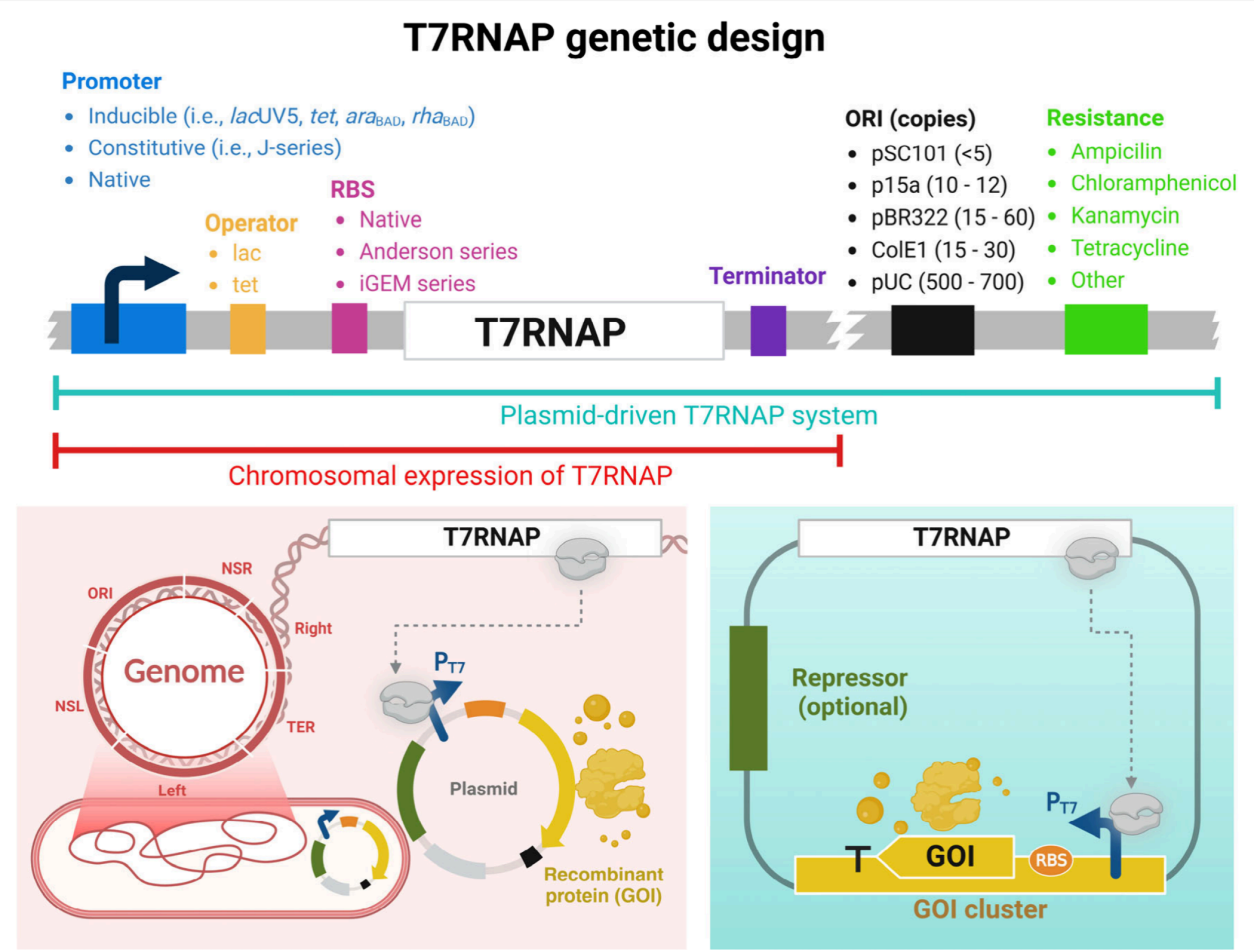
Genetic design of T7RNAP expression system in the chromosomal host (Upper) and the integration to chromosome for T7 promoter driving GOI expression (Pink box) or using a single plasmid-driven for the host-independent system (green box).

Orthogonality is one of the focuses between engineered gene circuits and host cells to perform new functions. Orthogonality can ensure the universal application of these genetic elements in different hosts by avoiding unnecessary crosstalk between the embedded elements in gene circuits and the host. Accordingly, a high cognate between T7RNAP and T7 promoter is an ideal approach for generating a precise orthogonal system. The T7RNAP genetic circuit can be designed in chromosomal integration or plasmid expression systems (Figure 3).

### 3.1 Chromosome-Integrated T7RNAP (CIT7) System

The T7 expression system was first constructed in *E. coli* by W. Studier by combining BL21(DE3) strain and pET plasmid. T7RNAP encoding gene is integrated into the genome under *lac*UV5 promoter control with IPTG as an inducer, while T7 promoter-driven GOI is located on the plasmid with a merged operator (*lacO*) for the inducible transcription. Once expressed, T7RNAP specifically recognizes the T7 promoter, leading to the efficient transcription of GOI.^58−60^ Although the T7 promoter is closely related to the bacteriophage T3-promoter, T7RNAP only has high specificity for the T7 promoter.^61,62^ Moreover, due to the simplicity of genetic design, T7RNAP was broadly expressed in dozens of hosts. Yet, the engineering progress of the T7 expression system in eukaryotes has fallen behind compared to prokaryotes, since mRNA translation in eukaryotes requires post-transcriptional and cytoplasmic transport processes. Intriguingly, eukaryotes from the kingdom Protista, *Trypanosoma* and *Leishmania*, have been successfully equipped with chromosomal T7RNAP expression. This ability is attributed to their mRNA trans-splicing mechanism,^10,63^ which is rarely observed in other eukaryotic species.

Among T7 system compartments, Lac operon is the standard regulator used to control the orthogonality of the T7 expression system during protein synthesis. In *E. coli* lineages, Lac operon in Nissle 1917 (EcN) is discovered to be weaker, with a strong *lac*Z level. The correlation of Lac operon and the T7RNAP level in EcN was evaluated by integrating the T7RNAP circuit at lambda (ET7L) and HK022 (ET7H) sites. By comprehensively analyzing the central dogma and cell behavior, ET7L could minimize inducer usage to stimulate tyrosine ammonia lyase protein synthesis.^19^ This implies that the chromosomal integration site of T7RNAP also plays a crucial role in reprogramming T7 apart from the Lac operon strength. Ting et al. characterized the T7RNAP efficiency at different chromosomal loci (i.e., lambda, HK022, phi80, and 186) in *E. coli* W3110 with carbonic anhydrase as GOI. From the result, the lambda site was concluded as the optimal integration site of T7RNAP, consistent with the T7 regulation design in BL21 (DE3) and EcN.^19,64^ Nevertheless, due to strong reliance on genomic information and genetic editing tools, the chromosomal-integrated T7 system faces critical limitations for universal and convenient applications, especially noncanonical hosts of Cyanobacteria and microalgae.^10,65,66^ Hence, most of the CIT7 design is still restricted to a few bacteria with known genome databases, such as *E. coli*, *Pseudomonas putida*, *Shewanella oneidensis*, *Ralstonia eutropha* (also known as *Cupriavidus necator*), and *Vibrio natriegens* from Gram-negative^19,21,67−69^ and *Bacillus substilis* and *Rhodococcus opacus* PD630 from Gram-positive.^70,71^

### 3.2 Host-Independent Expression System (HITES)

Within the 1990s, Studier’s group had harnessed the T7 transcription system to the host-independent design by combining T7RNAP with GOI-driven T7 promoter into a single plasmid. It was unexpected that the result caused the death of the host cell.^58−60^ Origin replication (ori) of the plasmid might lead to an excessive expression of T7RNAP, thus depleting cellular resources, generating biological stress, and causing mutations.^72−74^ Later, a host-independent expression system (HITES) was successfully developed using two biocompatible plasmids (low-copy-number and high-copy-number plasmids). This design created an autonomous self-regulated T7RNAP expression system by combining mixed feedback control loops and cross-species translation signals, called the Universal Bacterial Expression Resource (UBER).^75^ The system starts with a cross-species priming promoter that initiates the T7RNAP expression as a positive feedback loop, while the negative feedback loop aims to prevent toxicity from excessive T7RNAP levels under TetR repressor control. The system in UBER design could ensure the T7 transcription system and function in cross-species bacteria from Gram-positive strains, including *Corynebacterium glutamicum* and *Bacillus subtilis*; also, Gram-negative strains such as *Pseudomonas putida*, *Sinorhizobium*, and *Tatumella morbirosei*.^75^ However, the tedious optimization of feedback loop strengths between two plasmids is still required before applying to a new or noncanonical host. Another limitation of low gene expression and uncontrollable design might have appeared, as the promoter used is a constitutive system. The constitutive promoter used in the UBER system is a 456-bp sequence of eukaryotic origin that contains many bacterial promoter-like elements that allow continuous expression of T7RNAP without external regulation. Although this design ensures a baseline transcription level, it is difficult to modulate expression dynamically.^75,76^

As a higher T7RNAP level is unfavorable, by limiting T7RNAP quantity through fine-tuned circuit design^76−78^ or mutation,^76,79−82^ HITES or PDT7 (plasmid-driven T7RNAP) in a single plasmid was successfully constructed. For instance, Liang et al. created a fine-regulating transcription system of T7RNAP by combining antisense RNA design, CAP site deletion, terminator substitution, and ribosome-binding site (RBS) design. Compared to the BL21(DE3) host, this HITES can efficiently express recombinant proteins in non-DE3 hosts, such as *E. coli* JM109, *Pseudomonas putida*, and *Sinorhizobium* TH572, indicating its universality and powerful application potential in different prokaryotic hosts.^10,72,75^ Such findings reaffirmed that the small amount of T7RNAP was sufficient for the high-level GOI production under the T7 promoter.^76,77^ To deeply understand the toxicity and instability effect of T7 orthogonality, Tan et al. constructed PDT7 under different constitutive J-series promoters at low and high replication origins, generated 16 designs, and further tested them in *E. coli*. Interestingly, the T7-equipped *E. coli* cells could survive even when the strong promoter-driven T7RNAP was used. Through comprehensive analysis, it was discovered that a mutation has occurred on the T7RNAP or T7 promoter to attenuate aggressive competition.^77,83^

In Gram-negative *Bacillus substilis*, a novel T7 transcription system, called T7-BOOST (the T7-based optimized output strategy for transcription), has been developed to support both CIT7 and PDT7 expressions. This design involves a two-module plug-and-play system under a mixed T7 promoter and the IPTG- or the xylose-inducing promoters with its operator fusion (*lac*O or *xyl*O operator, respectively), generating a chimeric T7 promoter P_*T7lac*_ or P_*T7xyl*_. Compared to using a single inducible promoter, P_*hy-spank*_ and P_*xylA*_, the T7-BOOST system exhibited minimal leakage expression of sfGFP and a wider dynamic range. This is attributed to the double repression of the two modules and the strong transcriptional activity of the T7 promoter.^84,85^ Moreover, T7-BOOST allows easy transfer of the T7 transcription system to any other *B. subtilis* strain in a plug-and-play manner, instead of using the traditional integration of linear DNA fragments.^70,85,86^

To date, the construction of the T7 expression system often relies on trial-and-error tasks to obtain the desired recombinant strain by constructing and transforming numerous plasmids.^83,87^ Moreover, a labor-intensive effort for random testing of various combinations is required to achieve the target T7 system. The critical range of T7RNAP activity is the key in advance for successful construction. Cui et al. established a customized assay method to characterize T7RNAP activity using expression elements from HITES. Defining the upper limit of initial T7RNAP activity (E_i_L) was discovered to be a pivotal factor for successful HITES construction. If the initial activity (E_i_) was lower than E_i_L, a competent HITES could be achieved in the corresponding host and vice versa. Subsequent experiments presented the remarkable expression of sfGFP and D-MIase proteins across 13 host strains, guided by EiL values.^14^

Although constructing HITES does not rely on genomic databases like the CIT7 design, many non-model organisms still could not utilize the complete function of the T7 system. In prokaryotes, one of the most possible reasons is the incompatibility between the chassis and the heterogeneous T7 system, thus affecting the regulation. Indeed, different organisms have distinct codon preferences and require host-specific optimization to achieve an efficient translation of T7RNAP, followed by its orthogonality function to the T7 promoter. Moreover, codon optimization can help ensure proper folding and reduce the aggregation of T7RNAP, which is crucial for its activity.^73,88^ Meanwhile, in yeasts, an orthogonal T7RNAP transcription system has been achieved; however, the T7RNAP-transcribed mRNA failed to translate into protein. This unresolved challenge is related to complex transcription mechanisms.^89^ Accordingly, a few studies have searched for more potential T7-like RNAP/promoter systems as a solution, but the extensive characterization and effectiveness are yet to be established.^57^ Although innovations in CIT7 and HITES systems have expanded the potential applications of T7RNAP, challenges in dynamic regulation, host compatibility, and scalability underscore the need for further refinement.

## 4 How to Fine-Tune the T7RNAP Regulation?

Modifying the expression and stability of T7RNAP can optimize the overall performance of MCF. Eventually, efficiency also can be achieved if rational genetic design principles can guide the construction process. Of many bacterial hosts, *E. coli* recorded numerous derivatives with fine-tuned T7RNAP as summarized in Table 1. The representative strategies to construct this genetic circuit are described in this section.

**Table 1:** Summary of *E. coli* (DE3) Derivatives Incorporating Different Fine-Tuned T7RNAP Designs

**Strains**	**T7RNAP remark**	**Genetic features**	**Ref**
ASIA	Reduce RNA level	Utilize stress-inducible LysY	(110)
BL21(DE3)::P_tet_/ P_rhaBAD_/ P_araBAD_	Fine-tuned induction	Substitute P_lacUV5_ with P_tet_/P_rhaBAD_/P_araBAD_	(109)
BL21(DE3) pLysS	Reduce expression	Express T7 lysozyme	(145)
C41(DE3)	Reduce expression	P_lacUV5_ mutation	(90)
C43(DE3)	Reduce expression	P_lacUV5_ and lacI mutation	(90)
C44(DE3)	Reduce affinity	P_lacUV5_, lacI, and T7RNAP mutation	(107)
C45(DE3)	Reduce affinity	P_lacUV5_, lacI, and T7RNAP mutation	(107)
Evo21(DE3)	Reduce RNA level	Truncated RNase	(140)
Lemo21(DE3)	Reduce RNA level	Control T7 lysozyme with rhamnose	(91)

### 4.1 Circuit Elements Engineering

**Figure 4:**
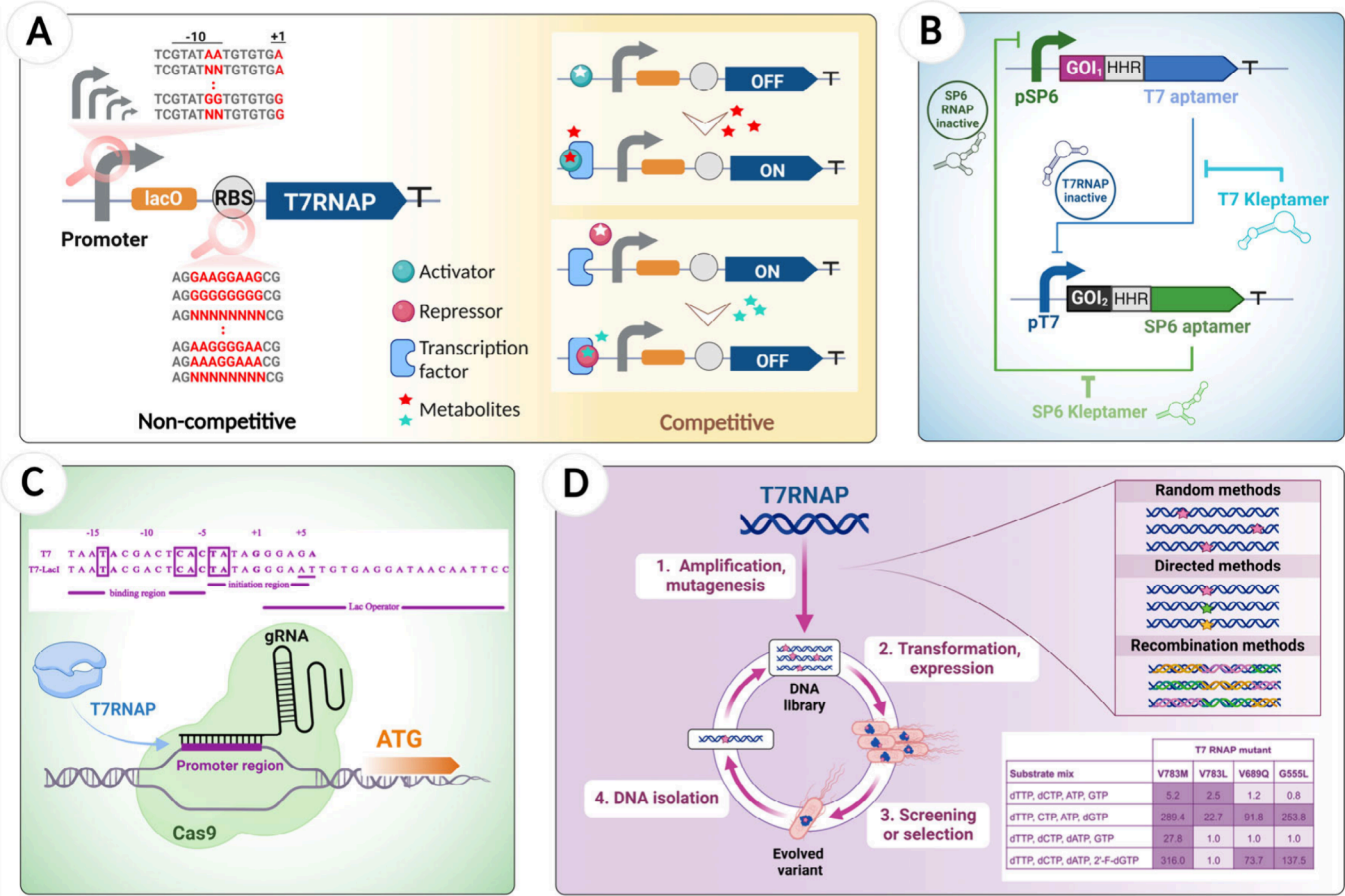
Representative of fine-tuning strategies in the T7RNAP regulation system. (A) Optimization of T7RNAP transcription and translation level, including substitutions of different promoters, promoter functional region, and RBS sequence in non-competitive and competitive designs. (B) RNA-based toggle switch design. This synthetic network produces two stable states that are reached by the mutual inhibition of the T7 and SP6 inhibitory aptamers. (C) CRISPR/Cas9 system utilizes dCas9 protein guided by a specific gRNA to bind to promoter regions, effectively enhancing and controlling T7RNAP binding. (D) Improvement of T7RNAP function via rational mutagenesis and directed evolution, e.g., increased the binding capacity to ribonucleotide triphosphate (rNTP) and deoxynucleotide triphosphate (dNTP) substrates.

Fine-tuning circuit elements of promoter and RBS is the first step in regulating T7RNAP transcription^78,84^ (Figure 4A). A series of BL21(DE3)-derived strains featuring the mutation in *lac*UV5 promoter and *yeh*U, C41(DE3) and C43(DE3), successfully depressed T7RNAP expression to maximize mitochondrial carrier protein 2-oxoglutarate production.^90^ In an inducible construct, LacI repressor protein is frequently employed in *E. coli* to respond to molecular signals for GOI expression. Previous work introduced two LacI binding sites with an approximate distance of 100 base pairs, successfully assisting LacI during DNA loop formation, reducing the accessibility of T7RNAP to the promoter, and improving repression efficiency.^91−93^ TetR is another repressor used to regulate gene expression that can be induced by tetracycline to allow transcription. LacI and TetR have been well-characterized for repression of T7 promoters in CFS and could achieve up to 10-fold repression.^91,94−97^ Aside from that, other inducible designs of the T7 system were developed using different chemical inducers (e.g., xylose, galactose, l-arabinose) to make it more applicable in laboratory research and industrial production.^10^ Recently, a large library of T7 promoter sequence variants has been quantified to assess promoter strength and adjust transcription activity. This library spans an activity range of over 4 orders of magnitude. Notably, a promoter sequence of T7Max was identified to exhibit stronger promoter strength than the native T7 promoter, expanding the upper limit of the T7RNAP transcription dynamic range.^54,95,98^ In recent years, a few software tools (i.e., PhiSITE, iProEP, and Promotech) have been developed to identify and analyze promoter regions, enabling precise control of transcription initiation. Among them, Promotech provides a large data set of promoter sequences from 9 distinct bacterial species, including those from Actinobacteria, Chlamydiae, Firmicutes, Proteobacteria, and Spirochaetes.^99−101^

In addition to the promoter, RBS has also aroused interest in improving orthogonality efficiency. In PDT7m (i.e., mutated T7RNAP in PDT7) vector, RBS also became the most contributing factor for enhancing the protein expression and aminolevulinic acid yield by 2.0- and 3.4-fold, respectively.^83^ The different expression strengths of T7RNAP can be obtained by changing the promoter and RBS sequences for different transcription and translation levels. Several algorithms have been developed to predict RBS strength, including the RBS Calculator,^102^ RBS Designer,^103^ UTR Designer,^104^ and EMOPEC (Empirical Model and Oligos for Protein Expression Changes).^105^ Using EMOPEC tool to modify a few bases of the Shine-Dalgarno (SD) sequence, the expression level of *E. coli* genes showed a 2-fold increment to the desired target. Moreover, the EMOPEC tool demonstrates design reliability up to 91% and better than using an RBS Calculator (47%).^102,105^ However, these tools are primarily based on data from *E. coli* and consider factors such as SD sequence, upstream and downstream nucleic acid sequences, mRNA secondary structure, and codon usage bias.

A robust library of fine-tuned T7 expression systems was developed in another fast-growth Gram-negative strain, *Vibrio natriegens*, with different promoters and RBSs upstream of T7RNAP. The variant VnDX-tet, in which the promoter of T7RNAP was changed from P_*lac*UV5_ to P_*tet*_, showed that the reporter gene of glucose dehydrogenase (GDH) activity was increased by 109% by the T7 expression system. Similarly, different T7RNAP translation levels were created by changing RBS sequences where the variant VnDX-RBS12/pGDH had the highest GDH activity with a 12.6% increment.^106^ Apart from promoter and RBS, harboring a stop codon within the T7RNAP gene (i.e., C44(DE3) and C45(DE3) strains) has presented unprecedentedly tight control of transgene expression, resulting in proper folding of membrane proteins during the stationary phase.^107^ Overall, fine-tuning promoter and the RBS sequence has proven crucial for optimizing T7RNAP transcription, enabling enhanced protein yields and precise gene expression. Despite advancements like stronger promoters, robust RBS designs, and innovative algorithms, challenges in expanding these tools beyond *E. coli* and ensuring universal adaptability persist, driving future innovation in synthetic biology.

### 4.2 Riboswitches and Quorum Sensing Signaling Pathways

Riboswitches and quorum-sensing systems are of heightened interest due to their capacity for autonomous regulation in a cell density-dependent manner, which circumvents the necessity for exogenous inducers and avoids any unwanted perturbation to the native metabolism of model hosts^108^ (Figure 4B). In particular, riboswitches can toggle between ON and OFF states in response to their cognate inducers. For instance, BL21(DE3) harboring pLysS plasmid for the T7 lysozyme, a natural inhibitor of T7RNAP, provides an efficient mechanism to inhibit the small amount of T7RNAP synthesized in the absence of inducer, due to its stochastic transcription from the *lac*UV5 promoter. However, leaky expression still occurs,^59^ thus triggering the development of swift tunable systems. Later, tight regulation of T7 lysozyme was developed by utilizing the P_rhaBAD_ system, generating Lemo21(DE3).^109^ Interestingly, substituting the P_rhaBAD_ with promoters from stress-induced proteins, known as ASIA design (Automated Stress-Inducible Adaptor), could tightly control T7 lysozyme expression, which then outperformed BL21(DE3) and original Lemo21(DE3).^110^ On the other hand, Dixon lab established the RiboTite system, consisting of BL21(IL3) strain (also known as the BL21[LV2] strain) and pETORS plasmid.^111−113^ BL21(IL3) possesses a similar configuration T7RNAP gene with BL21(DE3). In the pETORS expression plasmid, an orthogonal riboswitch sequence is contained in the 5′-untranslated regions of the T7RNAP gene and cloned into GOI upstream. Moreover, this riboswitch is a modified version of the adenine-sensing (i.e., *add* gene) A-riboswitch from *Vibrio vulnificus* which could bind to the effector pyrimido-pyrimidine-2,4-diamine (PPDA). As a result, the expression of the foreign coding sequence can only occur in the presence of IPTG and PPDA, where both inducers could also reduce the leaky expression and modulate recombinant protein level, respectively.^112−115^

Due to the highly efficient transcriptional characteristics of T7RNAP, utilizing its orthogonality system is expected to reduce the intracellular crosstalk to the native transcriptional network and serve as a signal amplifier. For instance, the quorum-sensing *LasR-P*_*lasI*_ circuit responsively managed the T7RNAP level and expressed GOI under the T7 promoter in *E. coli*.^91^ Since temperature is considered a versatile input signal, thermoresponsive genetic controls have gained significant interest in recombinant protein production and metabolic engineering applications.^116^ The previous study developed quorum-sensing (ThermoQS) circuits to trigger the prolonged expression of targeted genes under continuous heat exposure. The heat input was converted into quorum-sensing molecules of acyl-homoserine lactone derived from *Pseudomonas aeruginosa.* The acyl-homoserine lactone then activates the expression of T7RNAP and sustains the gene expression, allowing for temperature-dependent control of RNA synthesis. After heat treatment at 37 °C for 6 h, the ThermoQS design allowed *E. coli* to continuously express eGFP over 48 h which was 10-fold higher than that using the conventional operon of *cI*857*-P*_*R*_. Such a finding emphasized that the quorum-sensing controlled T7RNAP could sufficiently synthesize better than *cI*857*-P*_*R*_ regulation.^117^ Indeed, the development of riboswitch- and quorum-sensing-based systems underscores their utility in achieving efficient, autonomous control of T7RNAP, thereby overcoming the limitations of conventional regulatory approaches.

### 4.3 CRISPR-Mediated Gene Systems

Derived from archaeal and bacterial immune systems, CRISPR/Cas endonucleases have been exploited as a versatile genetic toolbox, generating a repertoire of CRISPR editing, CRISPR interference (CRISPRi), CRISPR activation (CRISPRa), and CRISPR imaging.^118−120^ In the CRISPR/Cas9 system, Cas9 is directed by guide RNA (sgRNA) to DNA, giving rise to a double-strand break (Figure 4C). Although the mutations of two active sites at HNH (H840A) and RuvC (D10A) domains occur and abolish the activity of Cas9 (dCas9), it still retains its DNA-binding activity.^121^ In CRISPRi design, sgRNA is designed to target a promoter or open reading frame and the sgRNA-dCas9 complex hampers the binding of RNAPs to the target, thus blocking the transcription initiation to elongation.^122^ In contrast, when dCas9 is tethered to the RNAP ω subunit or transcriptional activators, more RNAPs are recruited and transcription is intensified, known as CRISPRa.^123^ For instance, when dCas9 is fused with the T7RNAP in *E. coli*, the resulting dCas9-T7RNAP up-regulates gene transcription due to the recruitment of T7RNAPs and the binding of sgRNA to T7 promoter.^123^

The orthogonality from T7-based expression of sgRNA could improve the efficiency of the CRISPR system in various organisms. As the gene editing efficiency of the CRISPR system was usually limited by the poor expression of sgRNA, introducing a T7RNAP/promoter orthogonal system in *S. cerevisiae* increased the expression efficiency of gRNA by 80-fold.^25^ The T7 system was also used to direct RNA interference (RNAi) in *Aspergillus fumigatus* and *Aspergillus nidulans*. This system requires an inducible T7RNAP expressing cassette and an apical membrane antigen (AMA1) based episomal RNAi plasmid. However, this silencing system is unstable and may not be applicable for generating RNAi libraries.^124,125^

The combination of CRISPRa/i extends the scope of DNA targeting into multilevel regulation. In this case, an orthogonal trifunctional CRISPR system was developed by combining transcriptional activation, transcriptional interference, and gene deletion (CRISPR-AID).^126^ In *S. cerevisiae*, the CRISPR-AID system enables genome engineering and regulation, including transcriptional regulation and genome-scale modifications. Although T7RNAP does not completely function in yeast, the modified T7RNAP (P266L) successfully included an SV40 nuclear localization signal and effectively improved guide RNA expression by 80-fold.^127^ Moreover, CRISPR-AID allows metabolic regulation in a modular, parallel, and high-throughput manner. The CRISPR-AID system was upgraded by combining array-synthesized oligo pools into a genome-wide system (named MAGIC), which is effective for high-throughput genotype-phenotype mapping, indicating its potential for studying quantitative traits.^126^ In either CRISPR-AID or MAGIC systems, the T7RNAP pool is essential. Considered to have enormous potential for strain improvement, the CRISPR/Cas system has been widely harnessed for metabolic engineering of prokaryotes and eukaryotes, such as *Cupriavidus necator*,^68^*E. coli*,^23,128,129^*Staphylococcus aureus*,^130,131^*Synechococcus elongatus* UTEX 2973,^132^*Yarrowia lipolytica*,^133^ and *S. cerevisiae*.^133^ Integrating T7RNAP with CRISPRi/a systems has revolutionized genetic engineering by boosting sgRNA expression and enabling high-throughput regulation, though host-specific optimization remains a challenge.

### 4.4 Directed Evolution and Rational Mutagenesis

Directed evolution has been considered pivotal in enhancing the substrate versatility and catalytic activity of T7RNAP (Figure 4D). Residues 739–770 are the promoter recognition loop in T7RNAP which allows T7RNAP to contact the promoter specifically.^7^ Mutations to these critical parts of promoters usually result in a substantial loss in promoter recognition. Aside from evolving this part, mutating in other domain regions such as fingers, palm, thumb, NTD or CTD can improve the T7RNAP activity, stability, and function,^57,134−138^ where some works are summarized in Table 2.

**Table 2:** Mutation Residues of T7RNAP for Enhanced Functionality

**Mutant point**	**Domain position**	**Function**	**Ref**
P266L, K378R, S430P, N433T, S633P, Y639L, H784A, F849I, F880Y	NTD, Fingers, Palm, Thumb	2′-Modified-nucleoside incorporation and thermostability	(57)
Y639V, S430P, N433T, E593G, S633P, V685A, H784G, F849I, F880Y	Fingers, Palm	Improved activity	(134)
C723S	Fingers	Stability (reduced homodimers)	(135)
S430P, N433T, G542V, S633P, H772R, H784S, F849I, F880Y	Fingers, Palm	2′-Modified-nucleoside incorporation	(136)
G47A, G-ins-884^a^	NTD, CTD	Increased 3′- homogeneity	(137)
S43Y	NTD	Reduced dsRNA	(138)
V426L, A702V, V795I	Fingers, Palm	Thermostability	(142)
T75Q, A83K, I109L, H205S, K206P, I281P, A327P, T375 K, D388E, L446F, C510Q, L534V, V567P, G618Q, K642R, M832F, D834E, S856T, A863P, A866K	NTD, Fingers, Palm, Thumb	Thermostability	(143)
I320L, I396L, F546W, S684A, G788A	Fingers, Palm, Thumb	Thermostability and reduced dsRNA	(144)

a Insertion of amino acids.

In 1995, the “Y639F” mutant of T7RNAP was identified as functionally similar to DNA polymerase, thus reducing the ability of polymerase to discriminate between rNTPs and dNTPs. Despite this, it could maintain the original promoter specificity and catalytic activity.^139^ The serine residue at position 641 (S641), close to the Y639 site, also potentially influences the distinction between dNTPs and rNTPs. The double mutants “Y639F, S641A” showcase a higher ability than the wild-type T7RNAP during full-length RNA synthesis and possess the capacity for full-length DNA products *in vitro*.^82^ Furthermore, among 77 amino acid point saturated mutagenesis, four mutation sites (V783M, V783L, V689Q, G555L) were identified inducing substrate-specific changes. Notably, the “V783M” mutant successfully synthesized transcripts containing dTTP, dATP, dCTP, and 2′-F-dGTP with an efficiency comparable to that of the control Y639F. The double mutant “V783M, V689Q” improved the transcription efficiency by three times compared to the control.^82^

To improve the efficiency of T7RNAP, the mutation site is not restricted to its domain. For instance, the Evo21(DE3) strain was developed using directed evolution where the key mutation involves a truncation in the *rne* gene (RNase E), an essential enzyme involved in RNA degradation. The truncated RNase led to increased mRNA stability and reduced the degradation of transcripts, which is particularly beneficial for the expression of toxic or difficult-to-express proteins.^140^ The mutation in Evo21(DE3) is similar to a mutation previously engineered into the commercially available BL21Star(DE3) strain. This similarity underscores the importance of RNA stability in improving protein production and reducing toxicity.

On the other hand, although IVT using T7RNAP is notably prevalent, T7RNAP is known to produce a variety of byproducts during transcription. For instance, the presence of dsRNA in therapeutic RNA synthesized by T7RNAP *in vitro* can disrupt physiological signaling pathways and trigger an innate immune response.^141^ Although dsRNA can be purified through chromatography steps after IVT, the efficacy of the purification process decreases as RNA length increases. Consequently, it is crucial to minimize the production of double-stranded RNA during *in vitro* transcription. Recently, a residue in CTD was identified as a solvent-inaccessible cavity that permitted the incorporation of additional amino acids. Although a reduced side chain at residue 884 could improve the 3′-end homogeneity of the nucleic acid product, it still preserved RNA yields similar to those of the wild type. Moreover, the “G47A, 884G” double mutant significantly reduced dsRNA impurities and increased the 3′ homogeneity of the IVT mRNA transcripts.^138^ Another way to minimize the presence of dsRNA in the final IVT mRNA product would be to use a thermostable T7RNAP. A thermostable T7RNAP was generated to reduce the production of dsRNA by diminishing 3′-extension, thus yielding a higher mRNA purity when compared to wildtype T7RNAP.^142−144^ In short, directed evolution and random mutagenesis are key strategies for improving T7RNAP’s efficiency, versatility, and application-specific performance.

### 4.5 Nutrient Adjustment

Nutrient conditions also critically control the cellular and metabolic environment of the strain, streamlining with global changes in T7RNAP levels and thus affecting the transcription efficiency. For instance, glucose can repress the expression of T7RNAP in certain engineered systems such as catabolite repression.^77,78^ High glucose levels inhibit intracellular concentrations of a signaling molecule cyclic AMP (cAMP), thus hindering its binding to the catabolite activator protein (CAP). Hence, when the cAMP level is insufficient, *lac*UV5 promoter could not be activated, leading to reduced transcription of T7RNAP and its target genes.^108^ Based on these previous studies of the catabolite repression effect, introducing 1% glucose to low-carbon source media (such as LB or TB) could partially reduce the basal expression of T7RNAP and lower the leaky expression level of the target protein in BL21(DE3) to a level similar to that in BL21(DE3) pLysS.^145^ On the other hand, the sensitivity to catabolite repression can be reduced in the presence of glucose by mutating the *lac*UV5 promoter.^146^ Switching from glucose to other carbon sources, such as galactose, can also evade the catabolite repression issue. Under this condition, the expression of T7RNAP will be controlled under a galactose-responsive promoter and activated only in the presence of galactose.^147^

Nitrogen and phosphate availability can also influence T7RNAP regulation indirectly through the overall metabolic state of the cell, ensuring the sufficient energy and resources needed. For instance, peptone, tryptone, and yeast extract provide a rich mix of amino acids, peptides, and other necessary building blocks for protein synthesis. However, a higher nitrogen content from urea (>4 M) could cause protein unfolding and activity denaturation of T7RNAP.^148^ Meanwhile, phosphate is essential for nucleotide synthesis, and its availability can impact the transcriptional activity of T7RNAP.^33^

Furthermore, the crystal structure analysis of T7RNAP reveals that magnesium supply is crucial for its catalytic activity. Mg^2+^ ions help stabilize the negative charges on the phosphate backbone which contribute to the structural stability of T7RNAP, ensuring proper enzyme function and efficient transcription.^149^ Certain metabolites, such as indoles, can dynamically regulate T7RNAP activity. Researchers have used rational design and directed evolution to create low transcriptional activity of T7RNAP variants without indoles. When indoles are present, these variants exhibit a significant increase in activity, with some showing up to a 29-fold increase.^33^

Recently, it was found that utilizing animal-free and endotoxin-free medium can ensure high purity and functionality of T7RNAP, thus maximizing mRNA yields and enabling the efficient synthesis of long RNAs.^150^ Therefore, producing T7RNAP in such a medium presents safer and more ethical bioproduction practices. On the other hand, T7RNAP is very sensitive to salt and showed decreasing activity as opposed to normal RNAP at a concentration of >0.05 M. This pattern was also observed in the presence of other salts such as KCl, NH_4_Cl, and NaCl, implying that the effect of salt on T7RNAP is a general effect rather than cation-specific.^151,152^ Optimizing nutrient conditions for T7RNAP activity should be considered when it aims to reduce leaky expression, improve mRNA and protein yield, avoid metabolic disruptions and produce high-purity products.

## 5 Beneficial T7 Systems Used for Microbial Cell Factories

T7 systems are highly beneficial for microbial cell factories due to their efficiency and specificity in gene expression. In *E. coli*, aside from BL21(DE3), BL21-AI is one example of its versatility. This strain uses an arabinose-inducible system to control T7RNAP expression. A small phage-derived inhibitor peptide, called Gp2 (∼7 kDa), allows growth-decoupled protein production, including complex, toxic, and membrane proteins with multienzyme pathways.^31^ The beneficial T7 systems in MCF have led to more remarkable progress, including high-volume and high-value chemicals biomanufacturing, industrial enzyme production, and pharmaceutical protein synthesis, of which the recent 5 years of progress is summarized in Table 3. The orthogonal system between chromosome-integrated T7RNAP in *E. coli* W3110 and GOI-driven T7 promoters allowed for the independent regulation of multiple pathways and effectively produced cadaverine to 14.1 g/L using a whole-cell system.^153^ Further work expanded the host capability by fine-tuning the binding efficiency between T7RNAP and T7 promoter for gene mutagenesis (called dT7-Muta), producing 0.51 g/L itaconic acid.^154^

**Table 3:** Recent Accomplishments of the T7 System Used in Microbe Cell Factories

**Host**	**Targeted product**	**Main strategies of the T7 system**	**Achievement remarks**	**Ref**
*E. coli* Nissle	p-Coumaric acid	Fine-tune chromosomal expression of T7RNAP	λ integration site had better efficiency than HK022.	(19)
*E. coli* BL21(DE3)	5-ALA	Utilize a plasmid-driven system and mutate T7RNAP with TTTT insertion.	PDT7m enhanced 340% 5-ALA titer.	(83)
*E. coli* W3110	Cadaverine	Integrate T7RNAP at different chromosomal loci (i.e., lambda, HK022, phi80, and 186)	The optimum titer of 14.1 g/L titer was obtained by integrating T7RNAP at lambda site.	(153)
*E. coli* W3110	Itaconic acid (IA)	Fine-tune the binding efficiency between T7RNAP and the T7 promoter for gene mutagenesis (dT7-Muta)	AT-rich codons in the downstream regions of the T7 promoter achieved the highest IA.	(154)
*E. coli* S17–3	Citramalate	Integrate T7RNAP at the locus of LdhA or 16 S rRNA (*rrsG*).	Produce 8.1 g/L citramalate.	(155)
*E. coli* MG1655	L-homoserine	Engineer a dual-functional system for C-to-T and A-to-G *in vivo* mutagenesis (T7-DualMuta)	Feedback growth inhibition was alleviated up to 8 g/L of L-homoserine.	(156)
*E. coli* BL21(DE3)	Proinsulin	Integrate T7RNAP in BL21 to improve insulin gene expression.	>96% purity of proinsulin was achieved.	(157)
*Bacillus substilis*	Lanthipeptide	Control T7RNAP under P_43_ and integrate with repressor factor LacI at the *amyE* site.	Expressed 2 human-microbiota-derived lanthipeptides	(84)
*Bacillus substilis*	Hyaluronic acid (HA)	(i) Use strong promoter P_43_ and integrate T7RNAP at the *amyE* site; (ii) Screen the chromosomal integration site; (iii) Mutate repressor factor LacI.	The fine-tuned design module eliminated the need for an inducer and produced 6.86 g/L HA.	(158)
*Bacillus substilis*	l-Fucose	Screen the optimum promoter and integrate T7RNAP at *aprE* site.	P_xylA_ driven T7RNAP produced 1.6 g/L L-fucose.	(159)
*Bacillus substilis*	2′-Fucosyllactose	Integrate T7RNAP into genome under D-xylose control	Knockout xylA-xylB to reserve d-xylose as the inducer for T7 RNA polymerase expression	(160)
*Vibrio natriegens*	Glucose dehydrogenase protein	Establish a robust library with different promoters and RBSs upstream of T7RNAP.	Coupling the generated RBS12 and P_tet_ increased T7 expression system by 109% compared to P_lacUV5_.	(161)
*Pseudomonas putida*	Nicotinate dehydrogenase protein	Integrate T7RNAP at the *vdh* site using CRISPR/Cas9.	Protein production increased by 3.6-fold compared to other inducible systems.	(162)
*Shewanella oneidensis* MR-1	5-ALA	Integrate T7RNAP and 5-ALA producing gene cluster into chromosome.	The 5-ALA titer was improved to 145-folds.	(163)
*Clostridium saccharoperbutylacetonicum*	Hbd1^a^	Integrate a codon optimized T7RNAP into genome under a lactose-inducible control.	The first construction of T7 system, possessing a activity of Hbd1 with 8.3-fold higher than the native.	(164)
*Corynebacterium glutamicum*	Trans-glutaminase	Integrate T7RNAP and LacI into genome.	The enzyme activity reached 37.64 U/mL which was higher than 1 U/mL	(165)

a NADP+-dependent 3-hydroxybutyryl-CoA dehydrogenase (Hbd1).

Another work from a nonmodel *E. coli* S17-3 recorded that integrating T7RNAP at the locus of lactate dehydrogenase (*ldhA*) and 16S rRNA (*rrsG*) could compete with commercial BL21(DE3) strain in producing citramalate, a bulk monomer for biodegradable polymer.^155^ The efficiency of the T7 system was utilized for designing T7-DualMuta, which successfully accelerated the directed protein evolution of L-homoserine transporter (*rhtA*) and alleviated growth inhibition up to 8 g/L l-homoserine.^156^ High-purity insulin (>96%) was successfully produced using *E. coli* BL21(DE3) with a yield of 254.5 ± 11.7 μg/mL proinsulin.^157^ The use of T7RNAP in *E. coli* cell factories has advanced rapidly, unlike in other strains. This is expected, as *E. coli* has well-characterized transcriptional and translational machinery that aligns efficiently with T7RNAP. Meanwhile, other bacterial and eukaryotic hosts face challenges such as non-native T7 promoter incompatibility, nontranslatable transcripts in yeast due to lack of 5′ caps and 3′ poly(A) tails, as well as the energetic burden of the T7 system.

Notwithstanding disparities, several works have highlighted the potential of T7RNAP in *Bacillus substilis*.^79,158−160^ On the other hand, the incorporation of the T7 system facilitated the efficient production of desired recombinant proteins, enzymes, or chemicals in various hosts such as *Vibrio natriegens, Pseudomonas putida, Shewanella oneidensis* MR-1, *Clostridium saccharoperbutylacetonicum*, and *Corynebacterium glutamicum*.^161−165^ Although the complete function of T7 regulation systems in yeast has not been achieved, a higher T7-transcribed mRNA was successfully exported in *S. cerevisiae* after improving nuclear membrane permeability with viroporin HIV-1, thus providing a positive direction for efficient protein synthesis in yeast.^166^ T7RNAP efficiently produces RNA molecules in yeast, including CRISPR gRNAs and mRNA vaccines.^127,167^*In vivo* mutagenesis-assisted T7RNAP accelerates development of high-producing yeast strains.^24,168^ In nonmodel yeast *Yarrowia lipolytica*, T7RNAP controls engineered pathways without disrupting native metabolism.^169^ T7 systems excel in microbial cell factories, providing high efficiency for applications from chemical production to protein synthesis. Beyond established *E. coli* applications, expanding to nonmodel hosts and eukaryotes offers promising opportunities for MCF advancement.

## 6 Challenges and Opportunities

T7RNAP has been a protein of interest for more than 50 years since its first completed genetic map. The utmost importance to note is that less can indeed be more when it comes to T7RNAP effectiveness. As discussed above, the numerous studies highlighted here show the progress made; however, unresolved challenges toward a T7RNAP-based expression system mainly remain the same, such as (i) leakage of unintended GOI, (ii) imbalanced TX-TL-FD (transcription, translation, and folding), and (iii) high energy demand and reduced growth. Indeed, leakage of unintended GOI under inducer absence can lead to a less sensitive T7RNAP system, growth defects, and metabolic burden. Classically, repressor proteins and fragmented T7RNAP could address this issue, and engineering synthetic promoters with regulatory elements is still expected to improve the tightness of control over T7RNAP systems.

For balancing the TX-TL-FD system, gradual induction can allow the cell to adapt and allocate resources more efficiently, instead of using general controlled induction. In addition, harmonizing the codon usage of GOI and utilizing either chaperone or fusing tags may reduce ribosome stalling and equalize the protein synthesis process. In the case of high energy, the cells divert resources at the expense of other cellular processes during the rapid and high-level transcription driven by T7RNAP. Hence, incorporating energy regeneration systems and fine-tuning the expression conditions, i.e., using lower inducer or optimizing induction time, can help mitigate high energy consumption.

Despite these challenges, the T7 system still functions robustly in broad living cells of prokaryotic and eukaryotic strains, along with cell-free systems. The successful integration of T7RNAP into yeast and its ongoing research would undoubtedly expand the T7 system toolkit available for other eukaryotes as the model industrial workhorse. On the other hand, T7RNAP is quite promising, with several exciting opportunities on the horizons: (i) cell-free biomanufacturing, since the scalable cell-free production of T7RNAP is gaining traction, especially for mRNA vaccine synthesis; (ii) *in vivo* mutagenesis, where the MutaT7 and the T7-DualMuta toolkit enable all possible transition mutations (C-to-T and A-to-G) within living cells simultaneously; (iii) continuous evolution, for instance, systems like T7ACE (T7 RNAP mutant-assisted continuous evolution) could synthesize single-stranded DNA instead of RNA, leading to targeted hypermutations. This method can rapidly evolve antibiotic resistance and improve metabolic pathways.

Certainly, artificial intelligence and machine learning (AI-ML) are an emerging area of T7RNAP interest. It has been intensively adopted to predict mutations that could enhance T7RNAP stability, specificity, or efficiency under different conditions along with a low metabolic burden. Beyond evolution methods, AI-ML designs balanced TX-TL-FD synthetic circuits using T7RNAP. They simulate T7RNAP–host interactions and various induction strategies. AI-ML analyzes large data sets (e.g., HiKER) from high-throughput experiments, accelerating the discovery of new T7RNAP variants and regulatory elements. These highlights indeed will further advance the versatility and power of T7RNAP in driving genetic diversity, promoting the evolution of desired traits in various organisms, and creating powerful microbial cell factories and biotechnology applications.

## 7 Conclusion

While T7RNAP-based systems present significant challenges in achieving precise regulation, resource balance, and energy efficiency, ongoing advancements show promising solutions on the horizon. From scalable cell-free biomanufacturing to enabling rapid evolution *in vivo*, T7RNAP offers unparalleled opportunities in biotechnology. The integration of artificial intelligence and machine learning can further accelerate innovations, making T7RNAP a cornerstone for building versatile microbial factories and advancing synthetic biology. Essentially, this review serves as a roadmap to navigate the evolving landscape of T7RNAP research and unlock its full potential. Future research must focus on resolving existing limitations while exploring cross-species compatibility to unlock the full potential of T7RNAP in diverse systems. Together, these efforts will solidify the role of T7RNAP as a transformative tool for industrial and scientific progress in the decades to come.
